# Weighted Markov chains for forecasting and analysis in Incidence of infectious diseases in jiangsu Province, China^☆^

**DOI:** 10.1016/S1674-8301(10)60030-9

**Published:** 2010-05

**Authors:** Zhihang Peng, Changjun Bao, Yang Zhao, Honggang Yi, Letian Xia, Hao Yu, Hongbing Shen, Feng Chen

**Affiliations:** a Department of Epidemiology and Biostatistics, Nanjing Medical University School of Public Health, Nanjing 210029, Jiangsu Province, China; b Center for Disease Control and Prevention of Jiangsu Province, Nanjing 210029, Jiangsu Province, China; c Applied Mathematics Department, Hohai University, Nanjing 210029, Jiangsu Province, China

**Keywords:** weighted Markov chains, sequential cluster, infectious diseases, forecasting and analysis, Markov chain Monte Carlo

## Abstract

This paper first applies the sequential cluster method to set up the classification standard of infectious disease incidence state based on the fact that there are many uncertainty characteristics in the incidence course. Then the paper presents a weighted Markov chain, a method which is used to predict the future incidence state. This method assumes the standardized self-coefficients as weights based on the special characteristics of infectious disease incidence being a dependent stochastic variable. It also analyzes the characteristics of infectious diseases incidence via the Markov chain Monte Carlo method to make the long-term benefit of decision optimal. Our method is successfully validated using existing incidents data of infectious diseases in Jiangsu Province. In summation, this paper proposes ways to improve the accuracy of the weighted Markov chain, specifically in the field of infection epidemiology.

## INTRODUCTION

Mathematical models of any natural phenomenon should rest on some basic knowledge of the phenomenon and the data collected to track and understand it. Many years ago, J.L.Doob had defined a “stochastic process” as the mathematical abstraction of an empirical process whose development is governed by probabilistic laws. It is important to note that the term “stochastic process” refers to the mathematical abstraction, model, or representation of the empirical process and not to the empirical process itself. During recent years, the theory of stochastic process has developed very rapidly and has found application in a large number of fields[1].

In particular, a class of stochastic processes termed Markov chains or processes has been investigated extensively. Markov chains are one of the richest sources of models for capturing dynamic behavior with a large stochastic component[2],[3]. It is of great importance in many branches of science and engineering and in other fields, including physics[4],[5], industrial control[6],[7], reliability analysis[8], optimality analysis[9], economics[10],[11], etc. The Markov chains theory is a method of making quantitative analysis about the situation in which the system transfers from one state to another, hence predicting future tendencies. This provides a basis for making strategic analysis.

In the field of medicine and public health, the occurrence, development and prognosis of a disease will inevitably be affected by external factors and the human body factors. As these factors are closely interrelated with one another, it is difficult to explain them in a structural causal model. However, it is the interdependent relation between these data that is the most important and useful characteristic of the research objectives[12]. Here, it will be an effective way for us to establish a dynamic model in time order according to the change law of the disease.

In the past, many scholars have applied the Markov chain theory to forecast the incidence of infectious diseases, and established some corresponding mathematical models. In this way, various types of infectious diseases can be analyzed and studied comprehensively using the Markov chain theory. Markov processes have been applied in the study of the AIDS[13]–[15], contraceptives[16], ecology[17], cancer[18] and other diseases[19],[20]. Depending on the particular conditions of each study, different methodologies have been used. At the same time, different Markov models have been used in biomedical data analysis, especially for epidemiology research[21]–[25].

In this paper we will look at the use of Markov models for forecasting and analysis in the specific field of incidence of infectious diseases. These methods of quantitative analysis enjoy wide popularity because they are less dependent on historical data, have comparatively high accuracy and extensive adaptability. However, this kind of forecasting analysis based on the traditional Markov chain theory is destined to have defects and flaws. The homogeneity of the Markov chain has yet to be proved. There is enormous difficulty associated with adjusting the transition probability matrix, and the accuracy of the forecast is affected by objective factors.

This article attempts to overcome all these difficulties, and to establish a mathematical model to forecast the infectious diseases based on the weighted Markov chain theory. The authors will both leverage the advantages of the traditional Markov chain theory, and using the correlation analysis approach and historical data, seek more in-depth analysis of the usual characteristics that exist in the occurrence of the infectious diseases. These characteristics include long-term trends, seasonal characteristics, periodicities, short-term fluctuations and irregular variations.

The remainder of the paper proceeds as follows. The method of sequential cluster is described in Section 2. In Section 3 we describe the idea of weighted Markov chains. Markov chain Monte Carlo (MCMC) methods are considered in Section 4. Section 5 presents an application using real data from Jiangsu Province, and Section 6 contains some concluding remarks.

## ONE-DIMENSIONAL SEQUENTIAL CLUSTER ANALYSIS

Cluster analysis involves techniques that produce classifications from data that are initially unclassified, and should not be confused with discriminant analysis, where the number of existing distinct groups and corresponding data are known. There are two basic ways to search for clusters. These two methods are differentiated and categorized as either hierarchical or nonhierarchical in nature[26]. A variety of hierarchical clustering techniques have been implemented and successfully used to analyze or cluster one-dimensional and high-dimensional data[27]–[29]. Based on the characteristic of infectious disease incidence data, this paper attempts to only use the one-dimensional sequential cluster analysis algorithm to measure off the incidence data by SAS software.

To classify the one-dimensional sequential samples, partition points in the sequential series of samples are identified and the samples are then divided into several sections. Each section is unique, and this kind of classification can be called partitioning. Fisher proposed an algorithm for the optimum classification, namely the optimum partition method. The basic idea is based on the variance analysis: to look for a partition which can achieve minimum difference between the samples in the same section, and maximum difference between samples in some various sections. This is the optimum partition. Fisher suggests that the variation sections be divided by means of ordered cluster, and the data structure of the number of incidences can be fully taken into account so that the partition can be more reasonable.

Let any kind of variants *x*_1_, *x*_2_,…, *x_n_* be {*x_i_*, *x*_*i*+1_,…, *x_i_*}, *j* > *i*, *i*, *j* = 0,1,2...,*n* and define the mean vector 
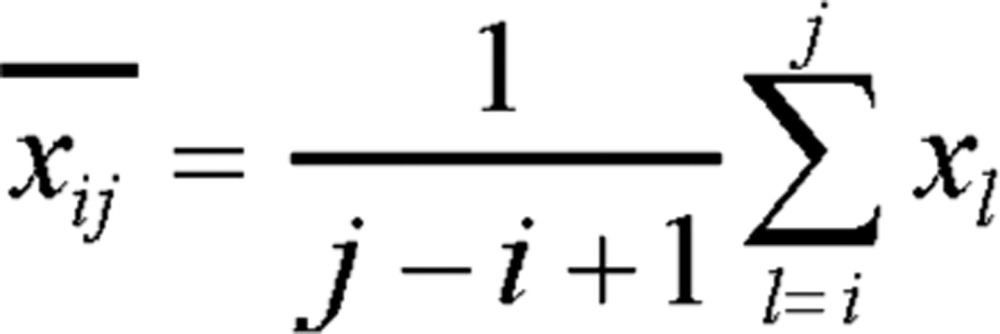
(1.1)

Define the total difference (the index is the sum of squares of deviations) of the samples in one kind as the diameter of that section, denoted as *D* (*i*, *j*): 
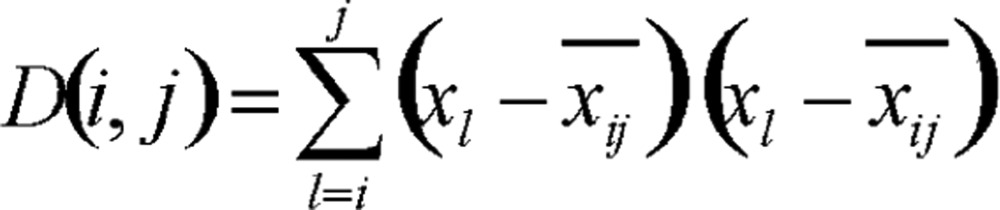
(1.2)

Divide *n* sequential variants into *k* kinds, and any partition can be

*P*(*n,k*):{*i*_1_,*i*_1_+1,…,*i*_2_-1},{*i*_2_,*i*_2_+1,…,*i*_3_-1},…,{*i_k_*,*i_k_*+1,…,*n*}

Define the error function, namely the objective function of this partition, and let it be the total sum of squares of deviations in this kind: 
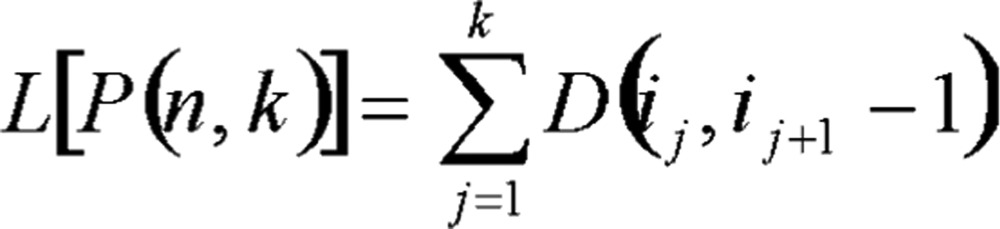
(1.3)

When *n* and *k* are fixed, the smaller the error function *L*[*P*(*n*,*k*)] is, the smaller the sum of squares of deviations within each kind, and this proves the reasonability of the classification. It can be proved that the so-called optimum partition is to make the *L*[*P*(*n*,*k*)] smallest. *k* can be calculated according to the relation curve of *L*[*P*(*n*,*k*)] and *k*. The value at the turn of the curve is the optimum partition number.

## WEIGHTED MARKOV CHAIN

A stochastic process *X*={*X*(*t*),*t*∈*T*} is a collection of random variables. That is, for each *t* in the *index set T*, *X*(*t*) is a random variable. We often interpret *t* as time and call *X*(*t*) the state of the process at time *t*. If *the index set* *T* is a countable set, we call *X*(*t*) a discrete-time stochastic process, and if T is a continuum, we call it a continuous-time stochastic process. The collection of possible values of *X*(*t*) is called state space. This general model has been described, from a theoretical analysis, by Chiang[30] and others[31].

### Markov chain

Markov chain is a branch of Markov process. If the present state of the system is given, then the past and future are (conditionally) independent. Such a behavior is called the *Markov property* of the system. A Markov chain evolves in a discrete (countable) state space with respect to discrete or continuous time.

A stochastic process *X*={*X*(*t*),*t*∈*T*} is defined on a probability space (Ω, F, P), where parameters set *T=*{0,1,2,…}, and state space *E*={0,1,2,…}. It is called a Markov chain if for any positive integers *l*,*m*,*k* and *j_l_* > … > *j*_2_ > *j*_1_ (*m* > *j_l_* ), *i_m+k_*,*i_m_*,*i_jl_*,…,*i_j_*_2_,*i_j_*_1_∈*E*, 
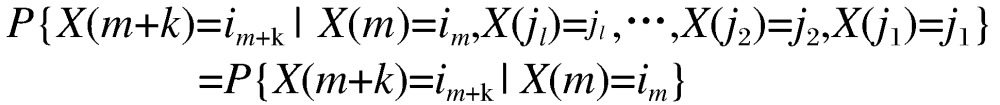
(2.1) For the aperiodic Markov chain, we have 
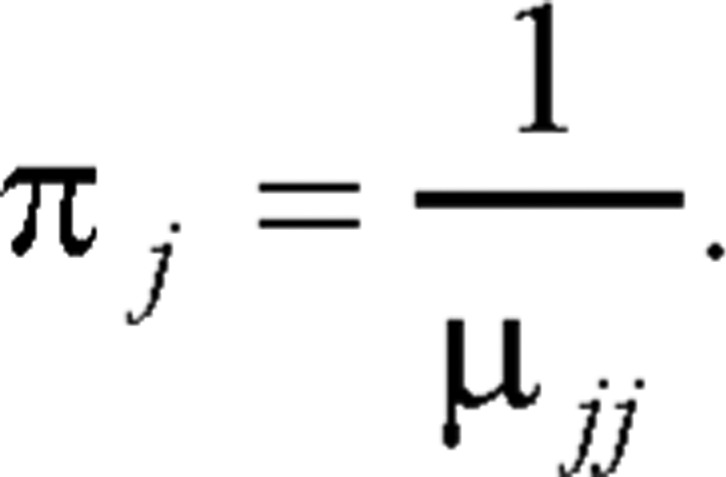
(2.2) where *µ_jj_* denote the mean recurrence time to state *j*, and *π_j_* is the limiting probability. The preceding identity shows that one way to find the limiting probability is by taking the reciprocal of the mean recurrence time. A simple way to find {*π_i_*} will be given shortly.

When an irreducible Markov chain is aperiodic and positive recurrent, the chain is called an *ergodic Markov chain*. The limiting distribution {*π_j_*} of an ergodic chain is the unique nonnegative solution of Equations: 
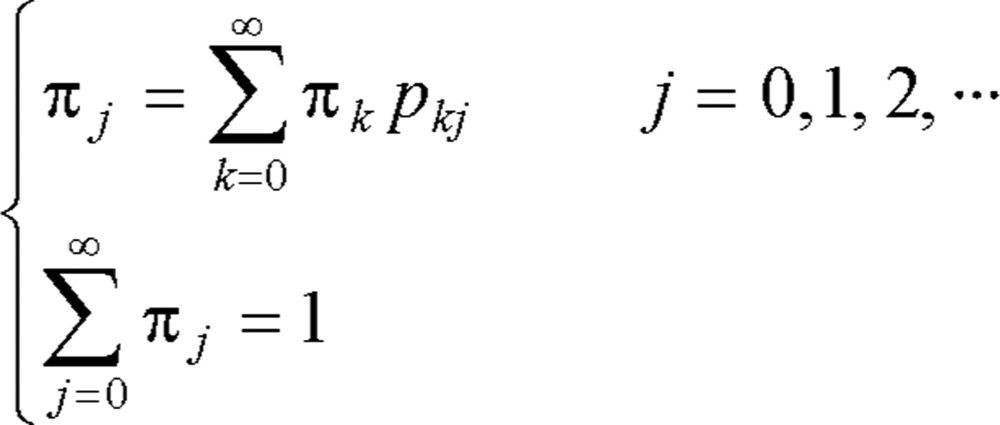
(2.3)

Now *π_j_* may be interpreted as the long-run proportion of time that the Markov chain is in state *j*. Thus it is easily seen to satisfy (2.2). The solution of these equations, sometimes, is not straightforward, and the MCMC methods may be used to solve them[32], which is considered in the next Section.

There are many properties and relative conclusions about Markov chain, and some other mathematical expressions (*e.g.*, *recurrent, limit theorems, periodic*, etc.) are described by Freedman[33] and Kendall and Montana[34].

### Weighted Markov chain

Because the monthly (or yearly, weekly) incidence of infectious disease are a series of correlative random variables, self-correlation coefficients depict various disease incidence data relationships. The past several months' incidence of infectious disease can be considered in advance to predict the present month incidence data. Then the weighted average can be made according to the incidence of the past several months infectious diseases compared with the present month's. Therefore the prediction purpose to make full and rational use of information is reached. That is the basic thought of weighted Markov chain prediction.

Based on the above discussion in this paper, the specific method of weighted Markov chain prediction is expressed as follows:

① Set up a classification standard of the monthly incidence of infectious disease according to the length of material series and the requirement of the specific problems. For instance, we can classify incidence of infectious disease as one-dimensional sequential cluster analysis in section 2 (corresponding to state space E={1, 2, 3, 4, 5,6}) and so on.

② Determine every month's incidence of infectious disease state according to the classification standard of “①”.

③ Compute various self-correlation coefficients *r_k_*, *k*∈E, 
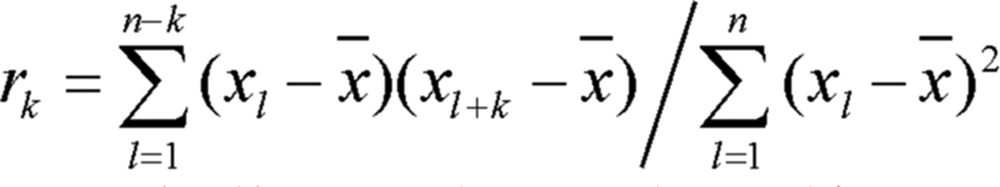
(2.4) where *r_k_* indicates *k* months self-correlation coefficient, *x_l_* (*l*=1,2,…,*n*) indicates the *l*th months infectious disease incidence, *x* indicates the mean value of *x_l_*, *n* indicates the length of monthly incidence of the infectious disease series.

④ Standardize various self-correlation coefficients. In other words, that is take 
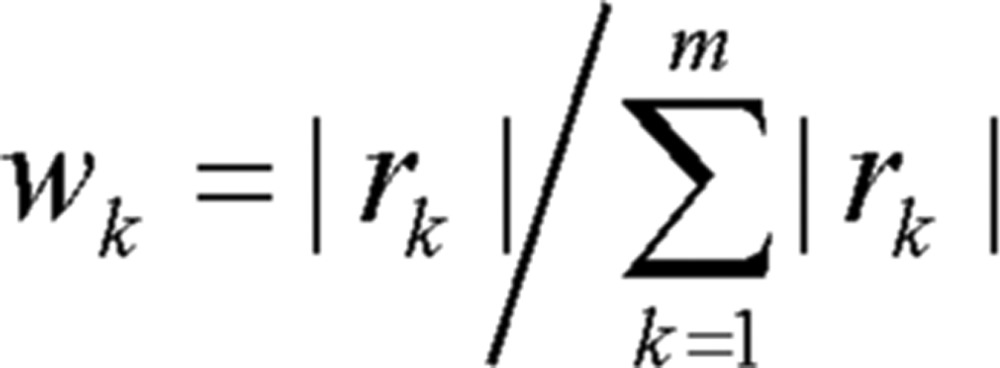
(2.5) as weights of various (steps) Markov chain (*m* is the maximum step according to prediction).

⑤ According to statistical results of “②”, we can get various steps of Markov chain transition probabilities matrixes, which decided the probability law when incidence of infectious disease states transited.

⑥ The past several months incidence of infectious disease can be initial states respectively, the state probability of the present month's incidence of infectious disease *P_i_*^(*k*)^,*i*∈*E* can be predicated and combined with relative transition probabilities matrixes, k indicates the step of Markov chain, k=1, 2, …, m.

⑦ Take the weighted average of various predicting probabilities of the same state as predicting probability of the plum rains intensity index, that is 
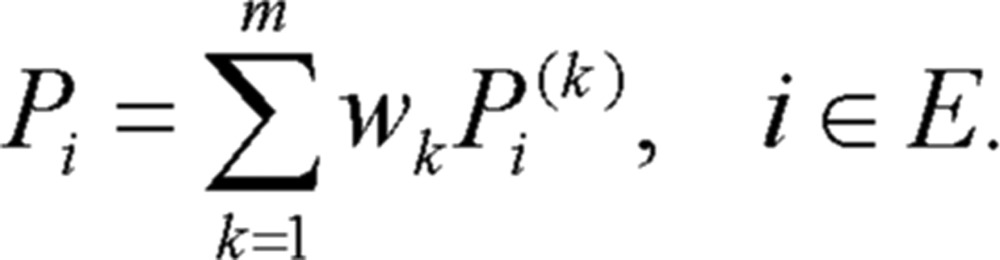
(2.6)

If *P_i_* =max{*P_i_*, *i*∈*E*}, *i* is the predicting state of the present month incidence of infectious disease. After the present month's incidence of infectious disease is determined, we can add it to the original series, repeating steps “①-⑦”, and the next month's incidence of infectious disease can be predicted.

⑧ The further analysis of Markov chain's characteristics (ergodic property, stationary distribution, etc.) also can be carried out[35],[36].

## MCMC METHODS

In this section we will describe MCMC methods for the weighted Markov chains. Our approach is analogous to the one used for solving the equations (2.3) in the previous section. Since there has been extensive research conducted and written about MCMC methods, we will be brief[37]. However, it should be noted that the full posterior distribution over all parameters in the model is unwieldy.

One standard method for constructing a Markov chain with the correct limiting distribution is via a recursive simulation of the so-called full conditional densities: that is, the density of a set or block of parameters. Each of the full conditional densities in the simulation is then sampled either directly (if the full conditional density belongs to a known family of distributions) or by utilizing a technique such as the Metropolis-Hastings (M-H) method. An important and crucial point is that these methods do not require knowledge of the intractable normalizing constant of the posterior distribution.

In the present case, we applied MCMC methods to solve the above equations (2.3), iterative and computational details are described in the recent papers of Chib and Winkelmann[38] and Covington *et al*[39].

## APPLICATION

In order to explain specific applications of this method and to conduct testing, this research is based on the samples of the monthly surveillance data of Hepatitis B patients in the period of January 1980 to October 2006 in Jiangsu Province. The weighted Markov chain theory was used to make a forecast and other related analysis of the incidents of the disease in November and February 2000.

Liver cancer is one of the most life-threatening cancers, and is the third-leading cause of death from cancer in China, and the top leading cause in the Province of Jiangsu. There are some 260,000 new cases of liver cancer each year throughout the world. Of all these cancer sufferers, about 42.5% are from China, and 90% of all liver cancer patients have previously been infected by Hepatitis B virus (HBV). A collection of data we gathered and analyzed suggests that about 25% of all those infected with HBV will eventually die of chronic severe hepatitis, cirrhosis of liver and liver cancer. Moreover, both acute and chronic Hepatitis B patients are the main source of infection for HBV. China is densely populated with Hepatitis B patients. According to a nationwide hepatitis epidemiological survey conducted in 2004, the average HBV infection rate of China is 70%-90% (including people infected and being infected). Therefore, the forecasting research of the incidence of HBV has far-reaching implications.

**Fig. 1:**
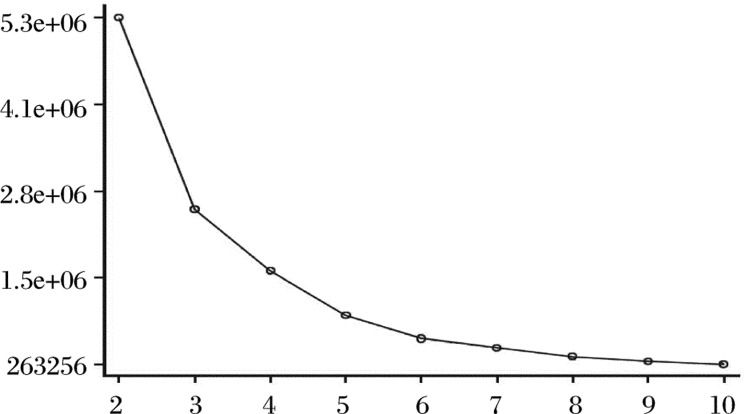
L[P(n, k)]∼k curve

Our forecasting and analysis study is as follows:

① Set up a classification standard of the monthly incidence of infectious disease according to the one-dimensional sequential cluster analysis algorithm by SAS 9.1.3 software. The value at the turn of the curve is *k* = 4 (see, e.g., ***Fig. 1***).

② As ***Table 1*** shows, the incidence data of infectious disease can be classified into 6 grades (corresponding to 4 states of weighted Markov chain), so various months' incidence of infectious disease states can be determined.

③ According to the ***Table 1*** classification standard, various self-correlation coefficients and Markov chain weights of various steps can be computed (***Table 2***).

④ After statistical computation, various one-step transition probabilities matrices with step's length 1, 2, 3, 4, 5 and 6 respectively were constructed:

⑤ We took the infectious disease incidence of July 1999 - Dec 1999's series to predict the Jan 2000's infectious disease incidence state. The results are shown below in ***Table 3***.

⑥ As ***Table 3*** shows, max{*P_i_*, *i*∈*E*} = 0.3734, then *i* = 3, and the infectious disease incidence state of Jan 2000 is 3. Corresponding infectious disease incidence data x satisfies: 1369 < x ≤ 1641. The actual infectious disease incidence state of Jan 2000 in Jiangsu Province is 1390, and the intensity state is 3. The prediction is correct.

Similarly, the Aug 1999 - Jan 2000 month series can be used to predict the infectious disease incidence state for Feb 2000. This forecasting process is just a repeat of “①-⑤”. The prediction results are listed below in ***Table 4***.

⑦ Further analysis of this weighted Markov chain's characteristics can be carried out as in ***Table 5***.

From ***Table 5***, we may infer that the return period of the state j is *T_j_*. The return period of each state will be *T*_1_ = 17.14(months), *T*_2_ = 7.5(months), *T*_3_ = 4.14(months), *T*_4_ = 5(months), *T*_5_ = 3.43(months), and *T*_6_ = 13.33(months) respectively. Thus it can be seen that, according to the classifying criteria determined in this article, the state of the number of incidents of Hepatitis B is most probable to appear about 3.43 months per time on average, and at 0.2917 percentage rate. The state 3 is the second, about 4.14 months per time on average, and the percentage is about 0.2417. States 4 and 2 are much less probable than the above; and the state 6 and 1 are least probable to appear, about 13.33 and 17.14 months respectively, with percentages of 0.0750 and 0.0583, respectively.

**Table 1:** Classification of incidence of infectious disease for Jiangsu Province

State	Incidence interval
1	x≤1029
2	1029 < x ≤ 1369
3	1369 < x ≤ 1641
4	1641 < x ≤ 1777
5	1777 < x ≤ 2071
6	X > 2071

**Table 2:** The weights of various steps Markov chain and various self-correlation coefficients

*k* *r*_*k*_ and *w*_*k*_	1	2	3	4	5	6
*r*_*k*_	0.4145	0.36038	0.1122	-0.08095	-0.09406	-0.09895
*w*_*k*_	0.3570	0.3104	0.0967	0.0697	0.0810	0.0852


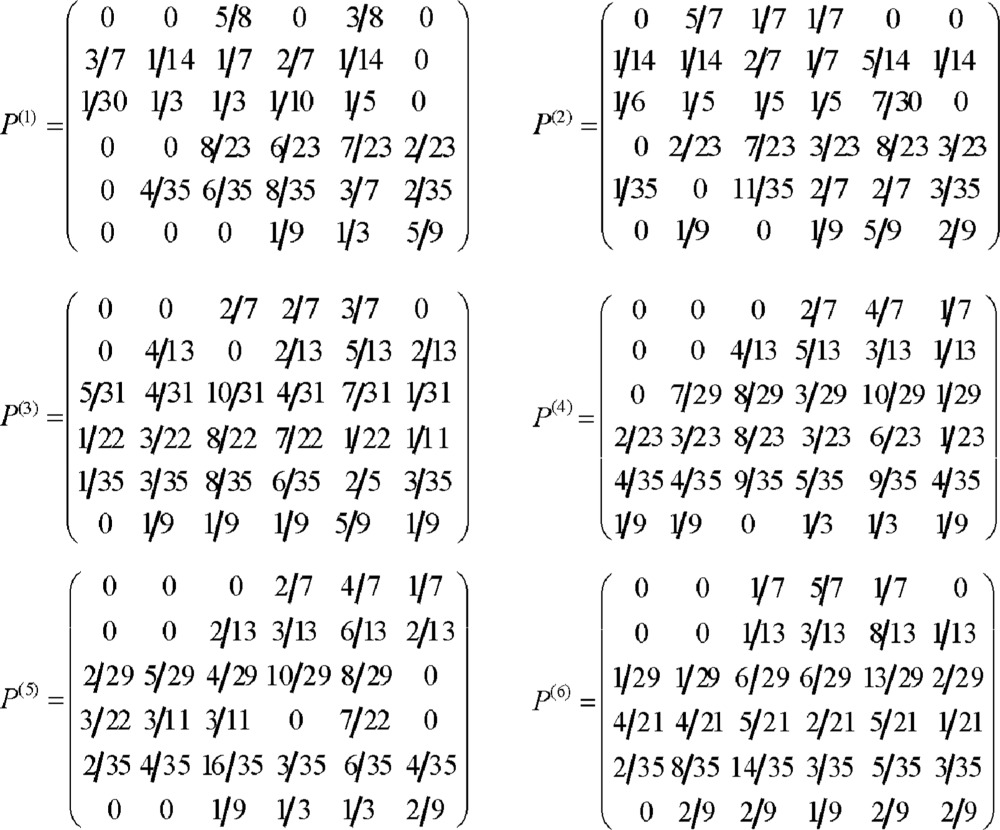


**Table 3:** Infectious disease incidence state prediction in Jan 2000

Initial year	State	Step (month)	Weight	State						Probability source
				1	2	3	4	5	6	
Dec 1999	1	1	0.3570	0	0	5/8	0	3/8	0	*P*^(1)^
Nov 1999	2	2	0.3104	1/14	1/14	2/7	1/7	5/14	1/14	*P*^(2)^
Oct 1999	2	3	0.0967	0	4/13	0	2/13	5/13	2/13	*P*^(3)^
Sept 1999	3	4	0.0697	0	7/29	8/29	3/29	10/29	1/29	*P*^(4)^
Aug 1999	4	5	0.0810	3/22	3/11	3/11	0	7/22	0	*P*^(5)^
Jul 1999	4	6	0.0852	4/21	4/21	5/21	2/21	5/21	1/21	*P*^(6)^
*P_i_* (weighted average)				0.0495	0.1071	0.3734	0.0745	0.3520	0.0435	

**Table 4:** Infectious disease incidence state prediction in Feb 2000

Initial year	State	Step (month)	Weight	State						Probability source
				1	2	3	4	5	6	
Jan 2000	3	1	0.3570	1/30	1/3	1/3	1/10	1/5	0	*P*^(1)^
Dec 1999	1	2	0.3104	0	5/7	1/7	1/7	0	0	*P*^(2)^
Nov 1999	2	3	0.0967	0	4/13	0	2/13	5/13	2/13	*P*^(3)^
Oct 1999	2	4	0.0697	0	0	4/13	5/13	3/13	1/13	*P*^(4)^
Sept 1999	3	5	0.0810	2/29	5/29	4/29	10/29	8/29	0	*P*^(5)^
Aug 1999	4	6	0.0852	4/21	4/21	5/21	2/21	5/21	1/21	*P*^(6)^
*P_i_* (weighted average)				0.0337	0.4007	0.2162	0.1578	0.1673	0.0243	

**Table 5.** Stationary distribution and recurrence period of various states

State(j)	1	2	3	4	5	6
*_πj_*	0.0583	0.1333	0.2417	0.2000	0.2917	0.0750
*T_*j*_*=*µ*_*j*_	17.14	7.5	4.14	5	3.43	13.33

## CONCLUDING REMARKS

The mathematical statistics tool is an important method for the prediction and forecast of infectious diseases. Historically, forecasting methods such as multivariate statistics analysis, Monte-Carlo simulations, spectrum analysis, that rely heavily on historical data have been used to infer future trends. But the accuracy of these non-subjective forecasting methods needs much improvement. In relation to these non-subjective forecasting methods, the weighted Markov chain theory introduced in this paper has the follow distinguishing characteristics:

① The key to the success of the forecast based on the weighted Markov chain theory in this article is the scientific classification, determination of the initial state of the system, and the ensuring of the state transition probability matrix. In contrast, previous forecasting methods have been heavily reliant on historical data, and largely affected by differences between historical and future environments.

② Since the weighted Markov chain is weighted with autocorrelation coefficient of various steps, the sum of the chain can be used to forecast the number of the infected. Therefore, it is more reasonable and sufficient in using data, and the Markov chain theory and the related analysis are well integrated. In the meantime, to calculate the limit distribution of the sequence applying the ergodic theorem reflects much more information of the sequence of the incidents of the disease in order to make a much more qualitative and quantitative description of the sequence calculated.

③ To determine the classifying criteria applying the ordered cluster, the data structure of the sequence of the patients can be taken full account of in the weighted Markov chain model, and the increase and decline in the historical data will be fully reflected. In this way, we are able to describe the status of the disease more accurately, so as to describe the internal distribution in a more effective way. Various methods in the multivariate statistics and the theory of fuzzy mathematics can be used to classify the state of the samples. The appliers should have a good understanding of the characteristics of the actual data, and accumulate experience in order to find more suitable classifying criteria.

④ With the continual increase of time sequence length, the representativeness of the historical data will be increased accordingly. The autocorrelation coefficient, transition probability matrix and the weight of various steps will change too, and this kind of change is also the process of improvement of the forecast and analysis theory. The forecasting model is not fixed, so the real number of the patients in every period of time should be added to the sequence of historical data. Therefore, the autocorrelation coefficient, transition probability matrix and the weight of the forecast can be adjusted online, and the accuracy of the forecast and analysis will be further improved. Moreover, the epidemic report of the disease forecast should have the same criteria in order to minimize the error and failure of reporting, and the disease information should be accumulated in the real practice.

⑤ With the development of the omy and culture, the improvement of hygiene conditions, and the strengthening of the prevention and control of epidemic diseases by the government, the epidemic diseases are controlled effectively, and the number of patients is declining year after year in China. In determining the structure of the model, all these changes should be paid attention to in order to make the statistical model more consistent with the life environment. Furthermore, as the number of the patients is able to reflect the change of the population and developing trend of the disease when the total population does not fluctuate too much, the paper applies the number of the patients to predict the future condition of the incidents of Hepatitis B in the coming year.

⑥ This forecasting method is effective when the spread and the prevention and control measures have not changed fundamentally. However, if preconditions are not met, the forecast will lose its value. Meanwhile, it is still challenging to calculate the actual number of the incidents of patients based on the state percentage calculated. It is very practical to see the occurrence and development of an epidemic disease as a stochastic process. The forecast and analysis method put forward in this article organically combines stochastic process theory, correlative analysis, ordered cluster analysis and epidemiology. Using an easy calculation and clear concepts, it provides a very good way to explore and discuss the forecast and prediction of epidemic diseases.
